# Hexokinase II as a Potential Target of Apigenin Through the Induction of Apoptosis and Necroptosis in Malignant Mesothelioma

**DOI:** 10.3390/nu18193276

**Published:** 2026-10-05

**Authors:** Moon-Kyun Cho, Sang-Han Lee, Hae-Seon Nam, Yoon-Jin Lee

**Affiliations:** 1 Department of Dermatology, Soonchunhyang University Hospital, Seoul 04401, Republic of Korea; mkcho@schmc.ac.kr; 2 Division of Molecular Cancer Research, Soonchunhyang Medical Research Institute, Soonchunhyang University, Cheonan 31511, Republic of Korea; m1037624@sch.ac.kr (S.-H.L.); namhs@sch.ac.kr (H.-S.N.); 3 Department of Biochemistry, College of Medicine, Soonchunhyang University, Cheonan 31511, Republic of Korea; 4 Department of Tropical Medicine, College of Medicine, Soonchunhyang University, Cheonan 31511, Republic of Korea

**Keywords:** Hexokinase II, apigenin, apoptosis, necroptosis, Warburg effect

## Abstract

**Background**: Lactate-acclimated MSTO-211HAcT and H2452AcT malignant mesothelioma (MM) cells were previously found to tolerate gemcitabine more readily than their parental cells while remaining sensitive to apigenin. We examined whether changes in glucose metabolism and HKII contribute to the apigenin response. **Methods**: Apigenin was evaluated in monolayer and spheroid cultures and in a xenograft model; HKII was also silenced in the acclimated cells. **Results**: Apigenin reduced cell viability, p-AKT (Ser473), glycolysis-associated proteins including HKII, and mitochondrial electron transport chain proteins, while increasing p53 and markers of cell death. Tumor growth and selected metabolic proteins were reduced in the xenograft model. HKII silencing reduced cell viability and glucose utilization, lowered cellular ATP, altered mitochondrial membrane potential, and increased markers of apoptosis and necroptosis. **Conclusions**: HKII-associated metabolism contributes to survival of lactate-acclimated MM cells, and apigenin affects this metabolic phenotype. Direct targeting of HKII by apigenin and the complete mitochondrial apoptotic pathway require further study.

## 1. Introduction

Malignant mesothelioma (MM) arises from the mesothelium lining the pleural, peritoneal, and pericardial cavities. Pleural disease accounts for most cases [1,2]. The 2015 global estimates included approximately 60,800 affected individuals and 32,000 deaths [3,4], while roughly 3000 new cases are diagnosed annually in the United States [5]. Exposure to asbestos is the principal recognized risk factor; genetic factors and simian virus 40 have also been discussed [6].

Treatment has expanded to include surgery, radiotherapy, systemic chemotherapy, and immunotherapy, yet MM remains difficult to detect early and long-term survival is limited. Cisplatin-based chemotherapy has been widely used, and treatment resistance continues to constrain outcomes [7]. Better understanding of the processes that sustain MM cell survival may help identify additional therapeutic approaches.

Many tumors use glucose through aerobic glycolysis even when oxygen is available, a pattern commonly called the Warburg effect. Besides supplying ATP, this pathway generates intermediates needed for biosynthesis [8]. PI3K/AKT, HIF-1α, p53, and MYC are among the regulators associated with this metabolic state [9]. Hexokinases (HKs) catalyze the first committed conversion of glucose to glucose-6-phosphate. HKII, one of four HK isoforms, is elevated in several cancers and has been linked to aggressive behavior [10,11,12]. Lactate can act as both a metabolic substrate and a signal within the tumor microenvironment and has been associated with treatment resistance [13]. MM cells can exhibit substantial glucose uptake on 18F-FDG positron emission tomography, providing a rationale for examining their glycolytic dependence [14,15].

Apigenin (4′,5,7-trihydroxyflavone) is a plant flavonoid present in foods including parsley, carrots, and celery [16]. Its reported anticancer actions span several cellular pathways [17], but its relationship to glycolytic metabolism in MM remains incompletely defined. In our earlier work, apigenin induced cell death and altered cell cycle progression in MSTO-211H and H2452 cells [18]. We subsequently found that lactate acclimation increased glycolytic features and gemcitabine tolerance, whereas apigenin retained activity in these cells; changes in AKT and p53 accompanied that response [19].

We therefore examined whether apigenin alters glucose metabolism and cell death in lactate-acclimated MM cells, using monolayer cultures, spheroids, and a xenograft model. HKII knockdown was included to evaluate the contribution of this enzyme to the observed cellular responses.

## 2. Materials and Methods

### 2.1. Cell Culture and Assays

The human malignant mesothelioma (MM) cell lines used in this study, MSTO-211H and H2452, were supplied by ATCC (American Type Culture Collection; Manassas, VA, USA). To establish lactate-acclimated populations, parental cells were cultured for 15 days (four consecutive passages) in RPMI-1640 medium (Welgene, Gyeongsan, Republic of Korea) supplemented with 3.8 μM lactic acid (Sigma-Aldrich, St. Louis, MO, USA). These populations are referred to as MSTO-211HAcT and H2452AcT. Both parental and acclimated populations were maintained as adherent monolayers. Complete RPMI-1640 comprised fetal bovine serum (5%), L-glutamine (1 mM), penicillin (100 U/mL), and streptomycin (100 μg/mL), supplied by Welgene (Gyeongsan, Republic of Korea). Cultures were kept at 37 °C in humidified 5% CO_2_ and allowed to equilibrate for 24 h before treatment.

For the viability experiments, cells were exposed for 48 h to 0, 10, 20, 30, 40, 50, or 60 μM apigenin (Sigma-Aldrich, St. Louis, MO, USA); vehicle controls received 0.1% dimethyl sulfoxide (DMSO; Sigma-Aldrich, St. Louis, MO, USA). Viability was measured by the MTT assay as described previously [18]. Hexokinase (HK) and pyruvate dehydrogenase (PDH) activities were determined with the HK Colorimetric Assay Kit (K789-100) and PDH Activity Assay Kit (K679-100), respectively, following the supplier’s instructions (BioVision, Milpitas, CA, USA). Glucose remaining in the culture supernatant was measured with the Glucose Colorimetric Assay Kit (K606-100; BioVision) to assess glucose utilization. Cellular ATP was quantified with the CellTiter-Glo^®^ Luminescent Cell Viability Assay (Promega, Madison, WI, USA), using a GloMax^®^-Multi microplate reader (Promega) to record luminescence.

### 2.2. Spheroid Culture and Viability Assay

Cells were seeded at 1 × 10^4^ cells per well in ultra-low-attachment 96-well plates (Corning Inc., Corning, NY, USA) and centrifuged at 500× *g* for 10 min to facilitate spheroid formation. Spheroids were grown for 5 days in complete RPMI-1640 medium (Welgene, Gyeongsan, Republic of Korea) containing lactic acid (Sigma-Aldrich, St. Louis, MO, USA). Formed spheroids were then treated for 48 h with 0, 10, 20, or 30 μM apigenin (Sigma-Aldrich, St. Louis, MO, USA).

Spheroid viability was examined by staining with fluorescein diacetate (FDA; 5 μg/mL) and propidium iodide (PI; 10 μg/mL), both from Sigma-Aldrich (St. Louis, MO, USA), to identify viable and non-viable cells. After washing with phosphate-buffered saline (PBS; Welgene, Gyeongsan, Republic of Korea), fluorescence images were acquired with a Leica EL6000 microscope (Leica Microsystems, Wetzlar, Germany). For quantitative assessment of spheroid viability, the protocol supplied with the Enhanced Cell Viability Assay Kit was followed (Young In Frontier Co., Ltd., Seoul, Republic of Korea). Cellvia reagent (10 μL) was added per well; after 1 h at room temperature, plates were gently mixed for 1 min and absorbance at 450 nm was measured on a GloMax^®^-Multi microplate reader (Promega).

### 2.3. Western Blotting

Cells were seeded at 1 × 10^5^ per well in 6-well plates (Corning Inc., Corning, NY, USA) and cultured in complete RPMI-1640 medium (Welgene, Gyeongsan, Republic of Korea) containing lactic acid (Sigma-Aldrich, St. Louis, MO, USA). After 48 h of treatment with 0, 10, 20, or 30 μM apigenin (Sigma-Aldrich, St. Louis, MO, USA), whole-cell protein was extracted in RIPA buffer containing 1× PBS (Welgene), 1% NP-40, 0.5% sodium deoxycholate, 0.1% SDS, and 10 μg/mL PMSF (the latter components from Sigma-Aldrich, St. Louis, MO, USA). Protein concentrations were determined using the Pierce^®^ BCA assay (Thermo Fisher Scientific, Frederick, MD, USA). Samples containing 40 μg protein were loaded onto 4–12% Bis-Tris NuPAGE gels supplied by Invitrogen (Carlsbad, CA, USA). After electrophoretic separation, proteins were transferred onto PVDF membranes from Cytiva Life Sciences (Marlborough, MA, USA).

Membranes were incubated at room temperature with the primary antibodies for 2 h. Detection used species-appropriate secondary antibodies conjugated to horseradish peroxidase (HRP). Bands were developed with enhanced chemiluminescence reagent (ECL; W1001; Promega, Madison, WI, USA) and captured on X-ray film. Membranes were stripped and reprobed for the corresponding total protein or β-actin when needed. Densitometry was performed with TINA 2.09 (Raytest, Straubenhardt, Germany). Signals for p-AKT (Ser473), p-MLKL (Ser358), and p-RIP3 (Ser227) were each divided by the signal for the corresponding total protein (AKT, MLKL, and RIP3, respectively); other target proteins were normalized to β-actin.

The antibodies and working dilutions were: OXPHOS cocktail (45-8199; 1:500; Invitrogen, Carlsbad, CA, USA); p-AKT (Ser473; 9271), AKT (9272), HKI (2024), HKII (2867), PFKP (8164), PDH (3205), p-MLKL (Ser358; 91689), MLKL (14993), p-RIP3 (Ser227; 93654), RIP3 (13526), Bcl-2 (2870), Bax (5023), PARP (9542), cleaved PARP (9541), caspase-3 (14220), and cleaved caspase-3 (9664), each at 1:500 (Cell Signaling Technology, Danvers, MA, USA); p53 (sc-126; 1:500), HRP-conjugated goat anti-rabbit IgG (sc-2004; 1:5000), and goat anti-mouse IgG (sc-2005; 1:5000) (Santa Cruz Biotechnology, Dallas, TX, USA); and β-actin (A2228; 1:1000; Sigma-Aldrich, St. Louis, MO, USA).

### 2.4. Xenograft Assay in Nude Mice

Approval for the mouse experiments was granted under protocol SCH20-0052 by Soonchunhyang University’s Institutional Animal Care and Use Committee. Five-week-old male BALB/c nude mice (22–24 g) were maintained in a specific pathogen-free facility; food and water were available ad libitum. Following a 1-week acclimation period, each mouse received a subcutaneous flank injection of 2 × 10^6^ MSTO-211HAcT cells in 50 μL serum-free medium mixed with 50 μL Matrigel (BD Biosciences, Franklin Lakes, NJ, USA).

Once tumors had formed, 12 mice were randomized into vehicle, apigenin, cisplatin, and apigenin-plus-cisplatin groups. Apigenin (20 mg/kg) and cisplatin (5 mg/kg), both from Sigma-Aldrich (St. Louis, MO, USA), were injected intratumorally three times weekly over 24 days. Animal condition, body weight, and tumor dimensions were monitored during treatment. At the endpoint, mice were euthanized by cervical dislocation and tumors were removed, measured, and stored at −80 °C for subsequent analyses. Tumor volume was calculated as follows:

Tumor volume (mm^3^) = length × width^2^/2.

### 2.5. siRNA-Mediated HKII Knockdown

For HKII knockdown, cells were transfected with HKII-targeting siRNA (siHKII; HSS179239) or Stealth RNAi negative control siRNA (siC; Oligo ID 452001), both from Invitrogen (Carlsbad, CA, USA). Cells were plated at 1 × 10^5^ per well in 6-well plates 24 h before transfection. At approximately 40% confluence, 25 pmol siRNA in 250 μL Opti-MEM (Gibco, Carlsbad, CA, USA) was combined with 7.5 μL Lipofectamine RNAiMAX (Invitrogen) for each well. Apigenin (30 μM) was added 24 h after transfection and maintained for a further 48 h. Cells were then collected for Annexin V staining, cell cycle analysis, Western blotting, or assessment of mitochondrial membrane potential.

### 2.6. Annexin V-PE/7-AAD Double Staining

Annexin V positivity and loss of membrane integrity were evaluated using the Muse kit for Annexin V and dead-cell detection (MCH100105; Merck KGaA, Darmstadt, Germany). Trypsinized cells were washed with PBS before a 30-min incubation with the Annexin V-PE/7-AAD staining reagent, protected from light and following the kit instructions. Stained cells were analyzed on a Muse cell analyzer (Merck KGaA).

### 2.7. Cell Cycle Analysis

Following transfection and a PBS wash, cells were kept overnight at −20 °C in 70% ethanol for fixation. The fixed cells were stained for 30 min in the dark with Muse Cell Cycle Reagent (MCH100106; Merck KGaA, Darmstadt, Germany), which contains PI and RNase. A MACSQuant analyzer (Miltenyi Biotec, Bergisch Gladbach, Germany) was used to acquire 10,000 events per sample, and cell cycle distributions were calculated with MACSQuantify software, version 2.5.

### 2.8. Mitochondrial Membrane Potential

To assess mitochondrial membrane potential, trypsinized and washed cells were resuspended at 1 × 10^6^ cells/mL and stained with 30 nM rhodamine 123 (Sigma-Aldrich, St. Louis, MO, USA). Staining proceeded for 30 min in the dark at 37 °C, after which the cells were washed to remove unbound dye. Fluorescence was acquired with a MACSQuant analyzer (Miltenyi Biotec, Bergisch Gladbach, Germany).

### 2.9. Reactive Oxygen Species (ROS)

Intracellular reactive oxygen species (ROS) were measured with 2′,7′-dichlorodihydrofluorescein diacetate (DCFH-DA; Sigma-Aldrich, St. Louis, MO, USA). For fluorescence detection, cells were incubated in the dark at 37 °C with DCFH-DA (10 μM) for 30 min and analyzed on a MACSQuant instrument (Miltenyi Biotec, Bergisch Gladbach, Germany). MACSQuantify software was used to analyze the data.

### 2.10. Statistical Analysis

The experiments were conducted independently on at least three occasions. Results are presented as means with their standard deviations (SD). Group means were compared by one-way ANOVA, with pairwise comparisons evaluated using Tukey’s post hoc procedure. Calculations were performed in SPSS 17.0 (SPSS Inc., Chicago, IL, USA), using 0.05 as the significance threshold.

## 3. Results

### 3.1. Apigenin Inhibited MM Cell Proliferation and Glucose Metabolism

**Figure 1:**
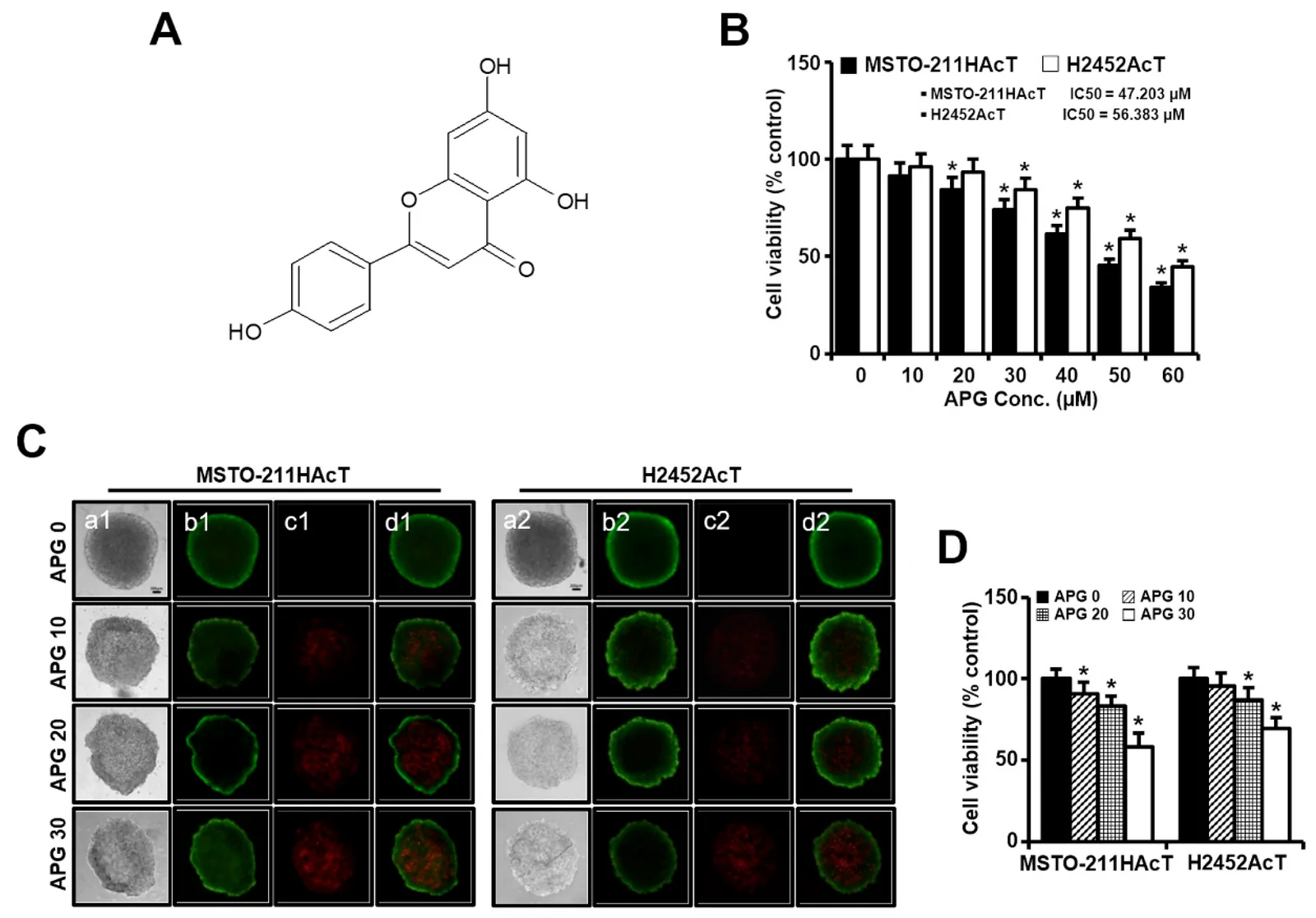
Effects of apigenin on the viability of lactate-acclimated malignant mesothelioma cells in 2D monolayer and 3D spheroid cultures. (**A**) Chemical structure of apigenin. (**B**) MSTO-211HAcT and H2452AcT cells were treated with apigenin (0–60 μM) for 48 h in RPMI-1640 medium containing 3.8 μM lactic acid. Cell viability was measured using the MTT assay. (**C**,**D**) Spheroids were treated with apigenin (0–30 μM) for 48 h in the same medium. (**C**) Representative images of MSTO-211HAcT (**a1**–**d1**) and H2452AcT (**a2**–**d2**) spheroids: phase-contrast images (**a1**,**a2**), fluorescein diacetate-positive live cells in green (**b1**,**b2**), propidium iodide-positive dead cells in red (**c1**,**c2**), and merged fluorescence images (**d1**,**d2**). (**D**) Spheroid cell viability. *p* < 0.05 versus the respective control group. APG, apigenin. Scale bar = 200 μm, The asterisk (*) indicates statistical significance at *p* < 0.05 versus the respective control group.

The chemical structure of apigenin is shown in Figure 1A. Apigenin reduced viability of MSTO-211HAcT and H2452AcT monolayers after 48 h over the tested 0–60 μM range. The estimated IC50 values were 47.2 and 56.3 μM, respectively (Figure 1B). In spheroids, apigenin reduced peripheral FDA fluorescence and increased central PI fluorescence (Figure 1C). After treatment with 30 μM apigenin for 48 h, measured spheroid viability was 58.2% of control for MSTO-211HAcT and 69.6% for H2452AcT (Figure 1D).

**Figure 2:**
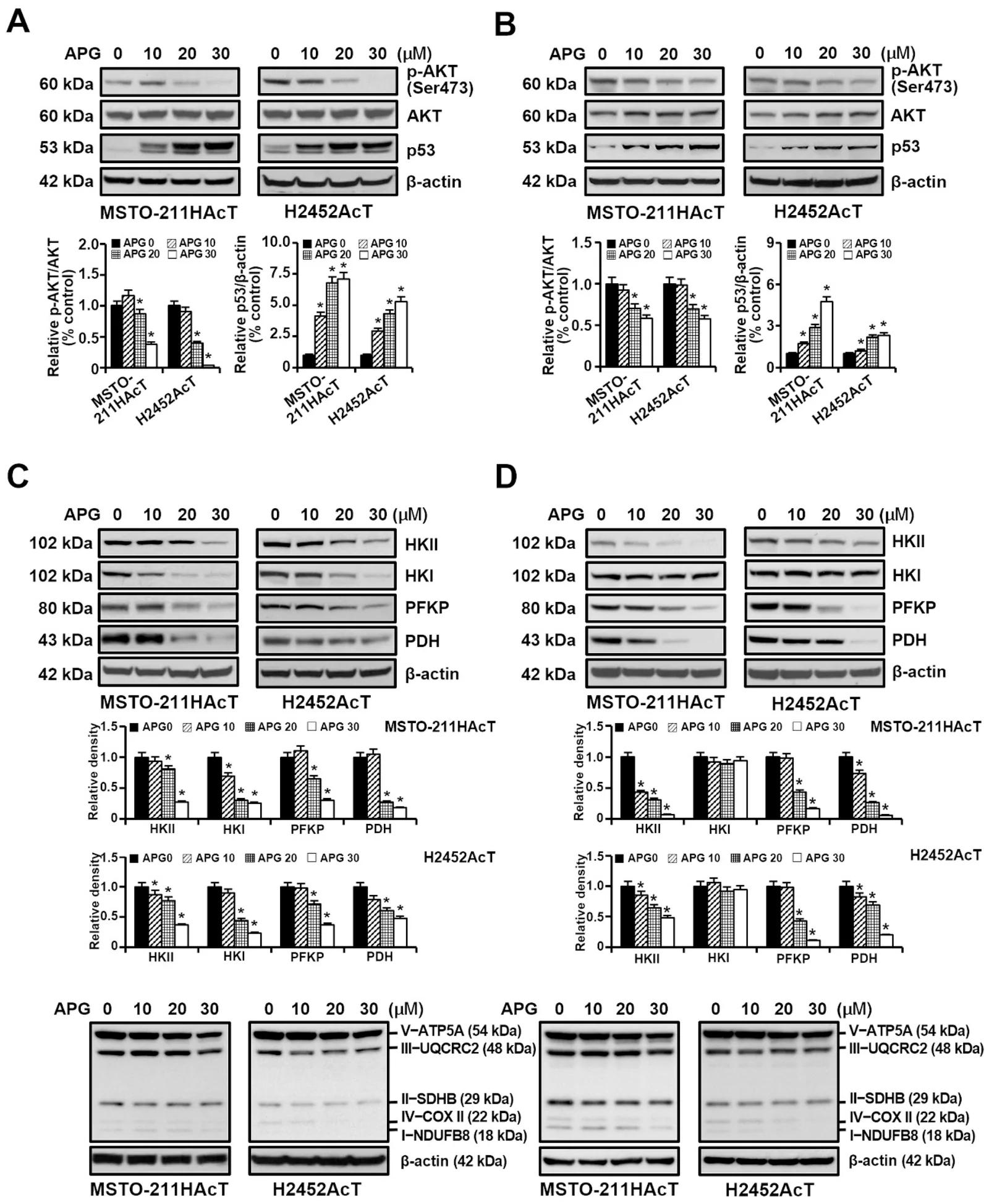
Effects of apigenin treatment on key proteins of glycolysis and mitochondrial electron transport chain in 2D monolayer and 3D spheroid cultures of lactate-acclimated malignant mesothelioma cells. (**A**) MSTO-211HAcT and H2452AcT cells were cultured in RPMI-1640 medium containing 3.8 μM lactic acid with or without different concentrations of apigenin for 48 h. (**B**) Spheroids were then treated with or without different concentrations of apigenin for 48 h in RPMI-1640 containing 3.8 μM lactic acid. Panels (**A**,**B**) show AKT signaling and p53; panels (**C**,**D**) show glycolysis-associated and OXPHOS proteins, measured by Western blotting. The plotted band intensities are expressed relative to β-actin for non-phosphorylated proteins; p-AKT (Ser473) was expressed relative to total AKT. * indicates *p* < 0.05 for comparison with the control for the same cell line or experimental model. APG, apigenin; HK, hexokinase; PFKP, phosphofructokinase platelet; PDH, pyruvate dehydrogenase; NDUFB8, NADH-ubiquinone oxidoreductase subunit B8 (complex I); SDHB, succinate dehydrogenase complex iron sulfur subunit B (complex II); UQCRC2, ubiquinone-cytochrome C reductase core protein 2 (complex III); COX II, mitochondrial cytochrome C oxidase subunit II (complex IV); ATP5A, ATP synthase F1 subunit alpha (complex V).

Western blotting was used to examine metabolic and signaling proteins in monolayer and spheroid cultures. Apigenin was associated with lower p-AKT (Ser473) and higher p53 levels in both cell lines under both culture conditions (Figure 2A,B). HKII, PFKP, PDH, and several OXPHOS proteins also decreased with treatment. HKI declined in monolayers but did not change significantly in spheroids (Figure 2C,D).

### 3.2. Apigenin Inhibits Xenograft Tumor Growth and Alters Metabolic and Cell Death Markers

**Figure 3:**
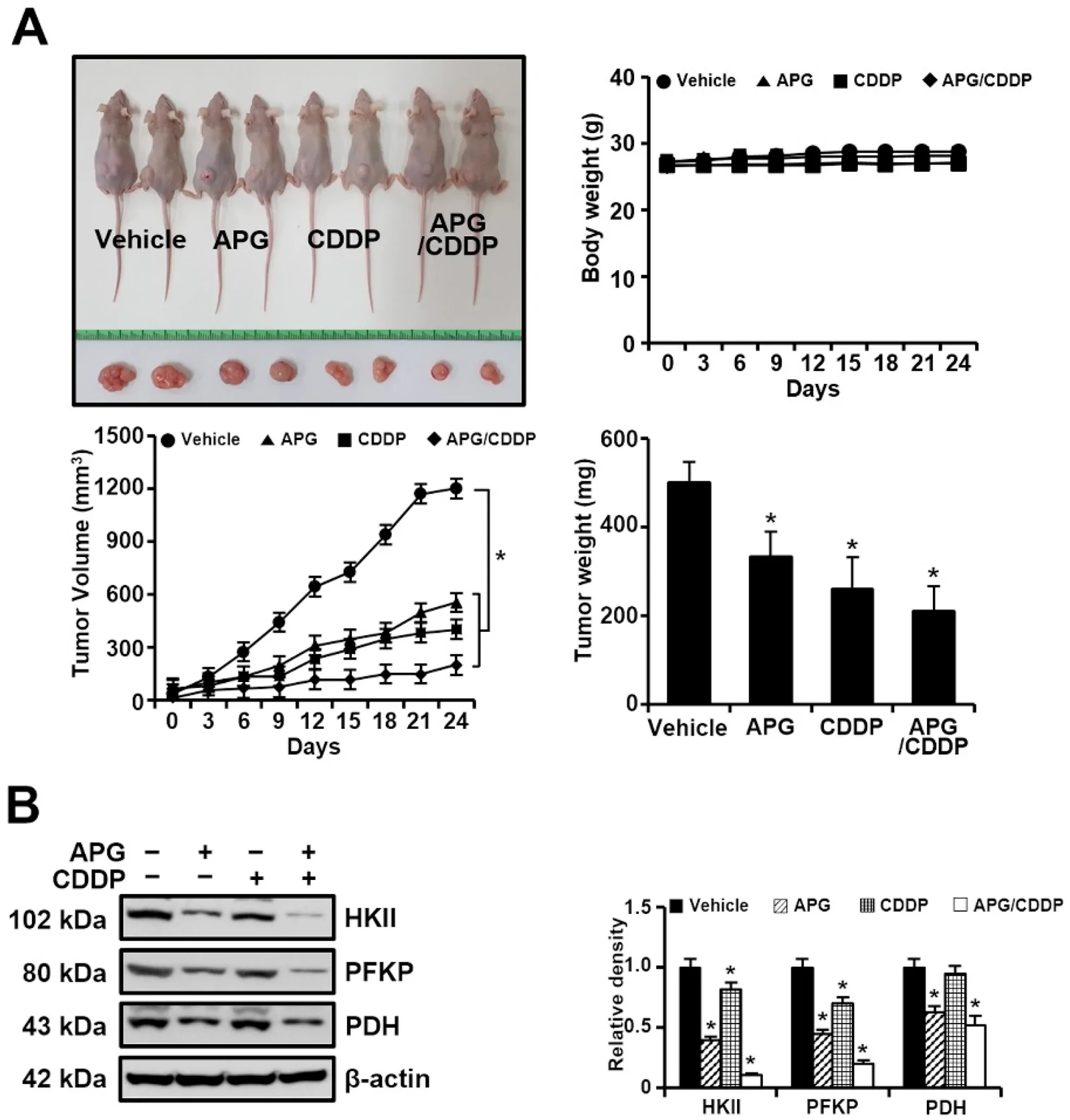

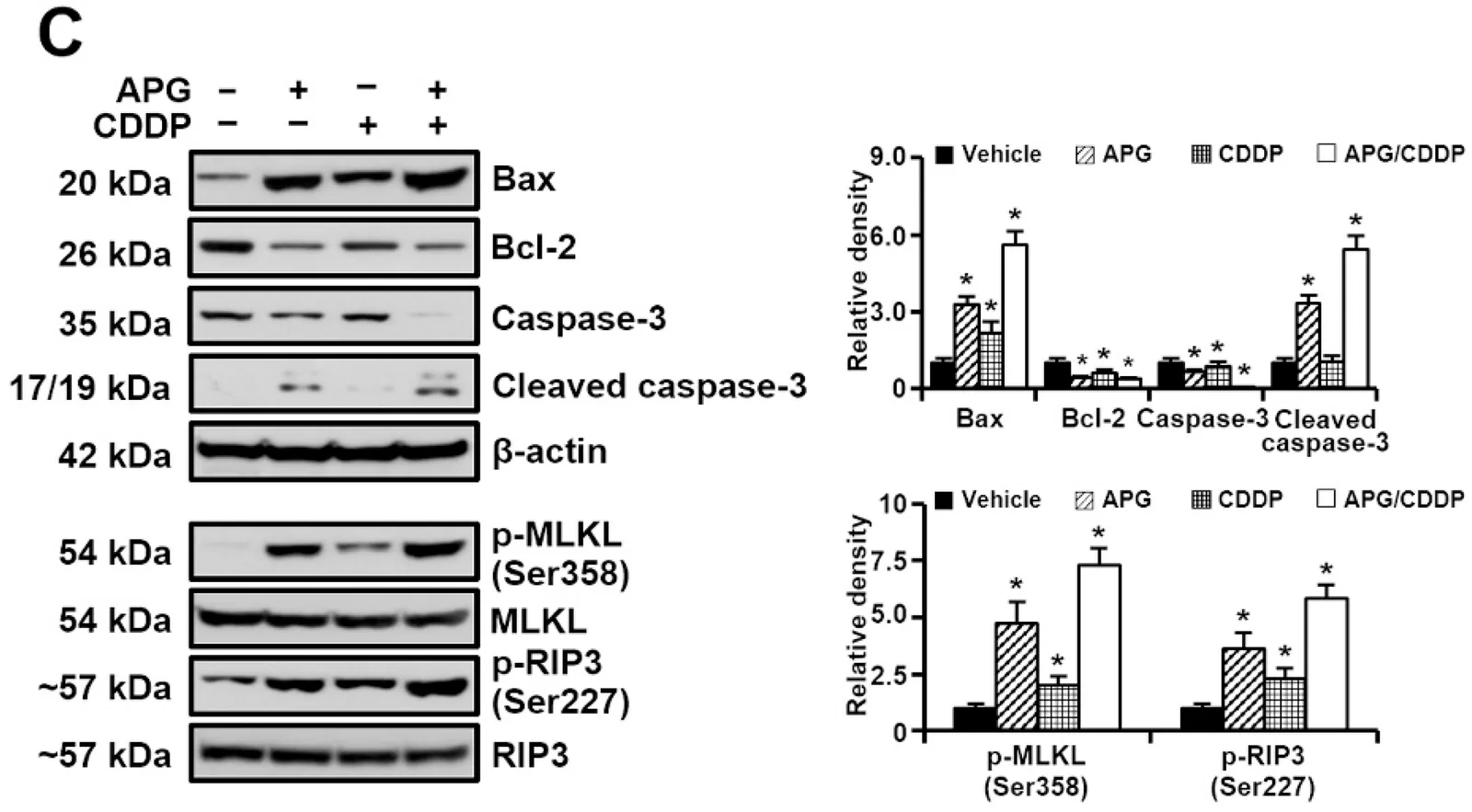
Effects of apigenin treatment in a nude mouse xenograft model of lactate-acclimated malignant mesothelioma cells. Mice were injected intratumorally with apigenin (20 mg/kg) and cisplatin (5 mg/kg), alone or in combination, three times per week for 24 days. (**A**) Representative mice, body weight, tumor volume, and tumor weight. (**B**) Expression levels of key regulatory enzymes of glycolysis. (**C**) Expression levels of marker proteins related to apoptosis and necroptosis. For densitometric quantification, p-MLKL (Ser358) and p-RIP3 (Ser227) signals were divided by total MLKL and RIP3, respectively. The remaining protein signals were divided by β-actin. * indicates *p* < 0.05 for comparison with the control for the same cell line or experimental model. APG, apigenin; CDDP, cisplatin; HK, hexokinase; PFKP, phosphofructokinase platelet; PDH, pyruvate dehydrogenase.

MSTO-211HAcT xenografts were used to examine whether the cellular response was reflected in tumors. Intratumoral apigenin (20 mg/kg) and cisplatin (5 mg/kg), given individually or together three times weekly for 24 days, reduced tumor volume relative to vehicle (Figure 3A). All mice survived the observation period, and no obvious change in body weight or general appearance was reported. Tumor lysates showed lower HKII, PFKP, and PDH in the apigenin-containing groups than in the cisplatin-only group (Figure 3B). Those groups also showed increases in the Bax/Bcl-2 ratio, cleaved caspase-3, p-MLKL (Ser358), and p-RIP3 (Ser227); the changes with cisplatin alone were smaller (Figure 3C). These markers support apoptosis- and necroptosis-associated responses but do not establish the upstream caspase-9 pathway.

### 3.3. HKII Knockdown Inhibits MM Cell Growth and Induces Apoptosis and Necroptosis

**Figure 4:**
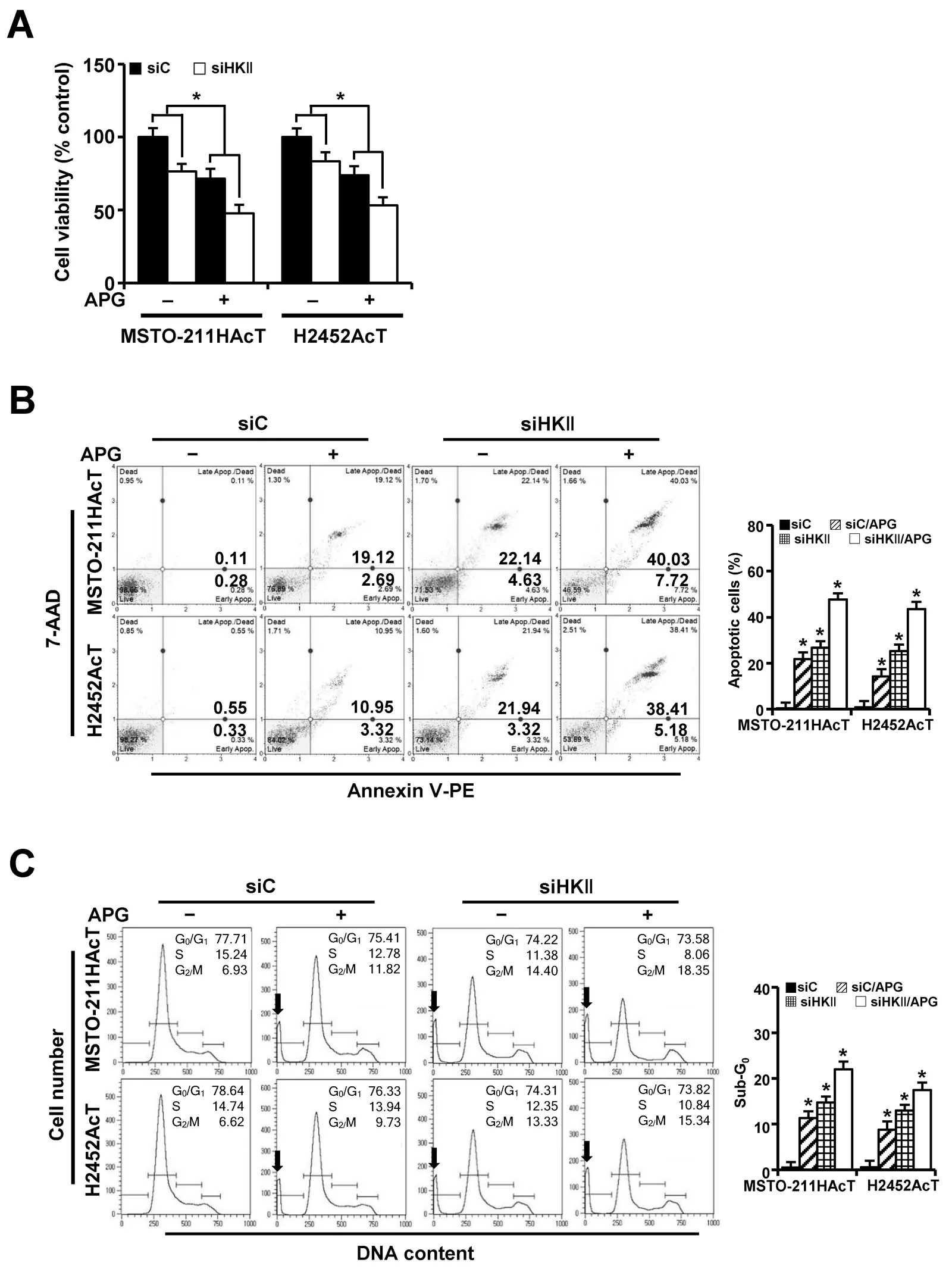
Effects of HKII gene silencing and apigenin treatment, alone or in combination, on viability and death in lactate-acclimated malignant mesothelioma cells. Following 24 h of transfection with HKII-targeting siRNA (siHKII, 10 nM) or the Stealth RNAi negative control (siC), cells received either 30 μM apigenin or vehicle for 48 h. The culture medium was RPMI-1640 supplemented with 3.8 μM lactic acid. (**A**) Percent cell viability. (**B**) Annexin V-PE binding assay. (**C**) Cell cycle analysis. * indicates *p* < 0.05 relative to the corresponding siC condition. Abbreviations: siC, Stealth RNAi negative control; siHKII, HKII-targeting siRNA; APG, apigenin. Arrows indicate sub-G_0_/G_1_ peaks.

**Figure 5:**
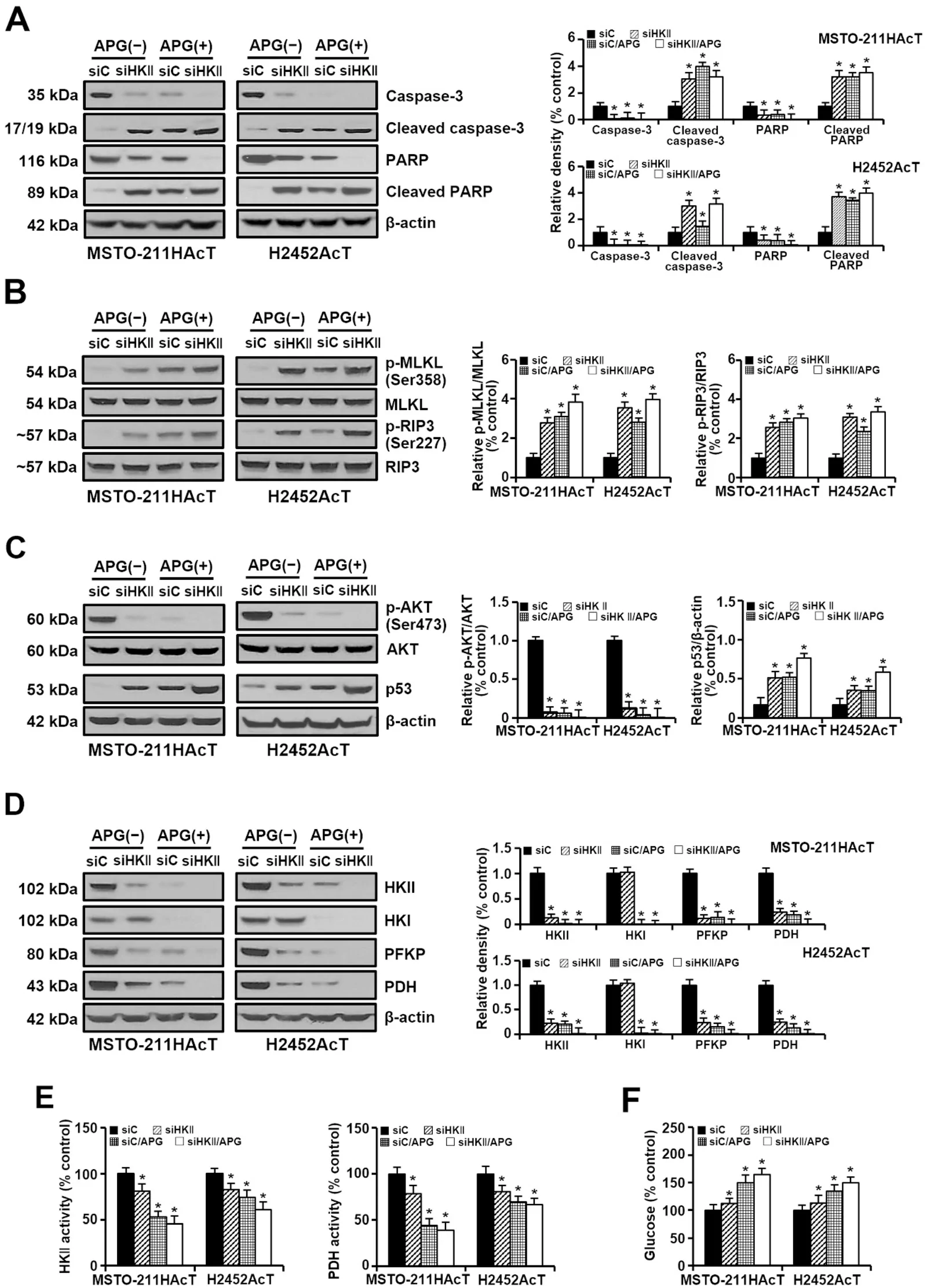
Effects of HKII gene silencing and apigenin treatment, alone or in combination, on key proteins of apoptosis, necroptosis, and glycolysis in lactate-acclimated malignant mesothelioma cells. Following 24 h of transfection with HKII-targeting siRNA (siHKII, 10 nM) or the Stealth RNAi negative control (siC), cells received either 30 μM apigenin or vehicle for 48 h. The culture medium was RPMI-1640 supplemented with 3.8 μM lactic acid. (**A**) Expression levels of apoptosis markers. (**B**) Expression levels of necroptosis markers. (**C**) Expression levels of p-AKT (Ser473) and p53. (**D**) Expression levels of key regulatory enzymes of glycolysis. (**E**) Activities of hexokinase and pyruvate dehydrogenase. (**F**) Changes in glucose concentration in culture medium. Densitometric values for phosphorylated AKT, MLKL, and RIP3 were calculated relative to their corresponding total proteins. β-actin served as the reference for the remaining targets. * indicates *p* < 0.05 relative to the corresponding siC condition. Abbreviations: siC, Stealth RNAi negative control; siHKII, HKII-targeting siRNA; APG, apigenin.

To assess the contribution of HKII, we transfected both acclimated lines with siHKII and evaluated apigenin treatment. Relative to siC, HKII knockdown reduced growth by about 23.6% in MSTO-211HAcT and 16.6% in H2452AcT; with apigenin, the corresponding reductions were about 52.3% and 46.8% (*p* < 0.05; Figure 4A). Annexin V-PE-positive cells and the sub-G0/G1 population increased (Figure 4B,C). Knockdown tended to reduce G0/G1 and S fractions and increase G2/M, with larger shifts after apigenin. Cleaved caspase-3 and cleaved PARP increased after HKII silencing (Figure 5A). Ratios of p-MLKL (Ser358)/MLKL and p-RIP3 (Ser227)/RIP3 rose by approximately 2.8- and 2.5-fold in MSTO-211HAcT cells, respectively, and by approximately 3.9- and 3.0-fold with added apigenin; H2452AcT cells showed a similar pattern (Figure 5B).

### 3.4. HKII Knockdown Depletes Cellular Energy Levels by Inhibiting Glycolysis and Mitochondrial Function

HKII silencing reduced p-AKT (Ser473) and increased p53, with larger changes when apigenin was added (Figure 5C). Relative to siC controls, HKII protein decreased by approximately 87% in MSTO-211HAcT and 78% in H2452AcT cells. PFKP and PDH protein levels also decreased, whereas HKI was not detectably affected by siHKII; apigenin further reduced HKII, PFKP, and PDH (Figure 5D). Measured HK and PDH activities were lower after HKII silencing (Figure 5E). Glucose remaining in the medium was approximately 12.0% and 13.3% higher, respectively, after silencing, and approximately 64.6% and 49.6% higher with the addition of apigenin, relative to siC controls (Figure 5F).

**Figure 6:**
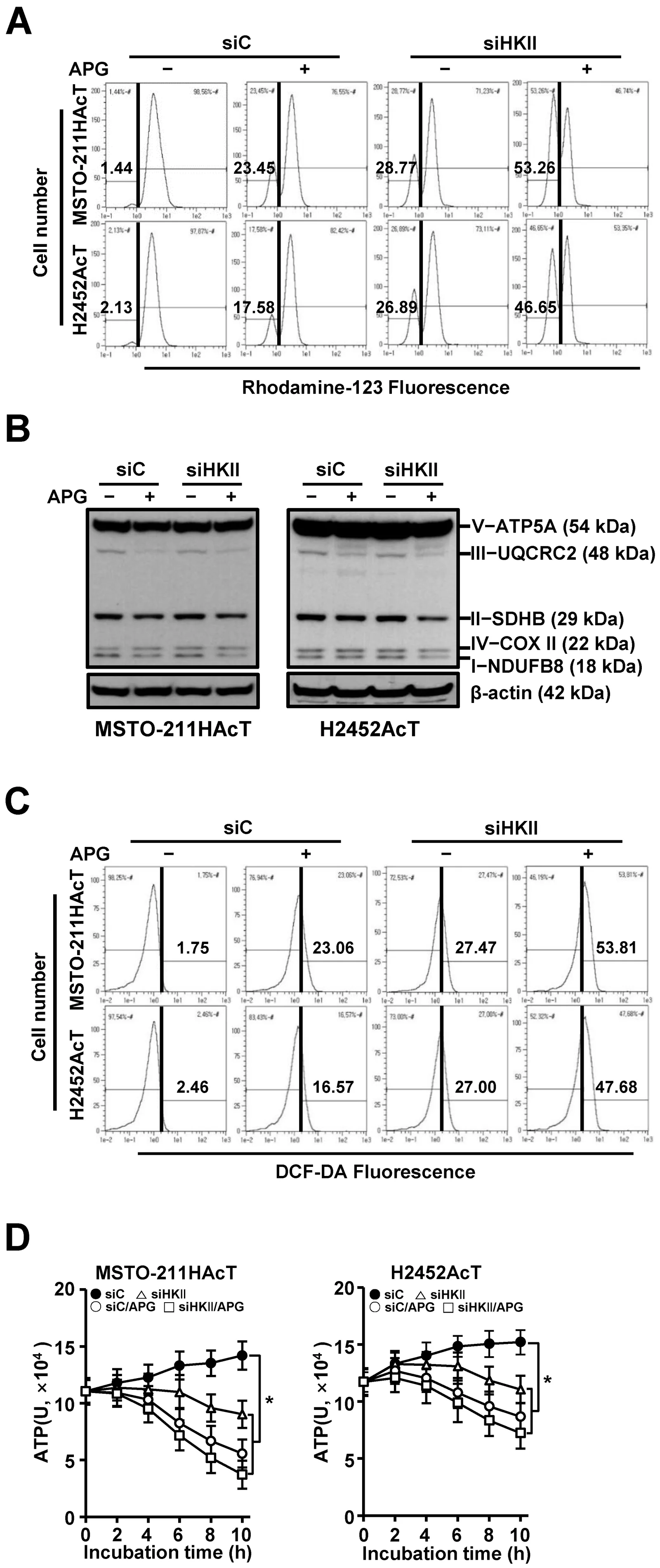
Effects of HKII gene silencing and apigenin treatment, alone or in combination, on mitochondrial function and ATP levels in lactate-acclimated malignant mesothelioma cells. Following 24 h of transfection with HKII-targeting siRNA (siHKII, 10 nM) or the Stealth RNAi negative control (siC), cells received either 30 μM apigenin or vehicle for 48 h. The culture medium was RPMI-1640 supplemented with 3.8 μM lactic acid. (**A**) Changes in mitochondrial membrane potential after staining cells with rhodamine123. (**B**) Expression levels of complexes I–V in mitochondrial electron transport chain. (**C**) Changes in intracellular ROS levels after staining cells with 2′,7′-dichlorodihydrofluorescein diacetate. (**D**) Changes in intracellular ATP levels. * indicates *p* < 0.05 relative to the corresponding siC condition. Abbreviations: siC, Stealth RNAi negative control; siHKII, HKII-targeting siRNA; APG, apigenin; OXPHOS protein abbreviations are defined in Figure 2. DCFH-DA denotes 2′,7′-dichlorodihydrofluorescein diacetate.

Rhodamine 123 staining indicated an increase in the depolarized mitochondrial fraction after HKII silencing, from 1.44% to 28.77% in MSTO-211HAcT and from 2.13% to 26.89% in H2452AcT; adding apigenin increased the shift further (Figure 6A). Apigenin lowered the measured OXPHOS complex proteins, whereas siHKII alone did not show the same protein changes (Figure 6B). The high-ROS cell fraction increased from 1.75% to 27.47% and from 2.46% to 27.0% in the two lines, respectively, following knockdown, with further increases in the combined-treatment groups (Figure 6C). ATP levels were highest in controls and lower after HKII knockdown, apigenin, and their combination, in that order (Figure 6D).

## 4. Discussion

The findings place HKII-associated glucose metabolism among the processes affected by apigenin in lactate-acclimated MM cells. The effects of HKII silencing partly overlap with those of apigenin, while the mitochondrial findings indicate that apigenin has effects beyond HKII depletion. Together, the cell and xenograft observations support further investigation of this metabolic vulnerability, although they do not establish HKII as a direct molecular binding target of apigenin.

Apigenin reduced the abundance of HKI, HKII, PFKP, and PDH, consistent with altered glucose metabolism. HK catalyzes phosphorylation of glucose to glucose-6-phosphate at the entry point of glycolysis; PDH acts downstream of glycolysis at the interface with mitochondrial metabolism. These proteins therefore should not all be described as enzymes of the same rate-limiting glycolytic step. Cancer cells use aerobic glycolysis to support proliferation and biosynthetic needs [8,20], and high HKII expression has been reported in several malignancies [21,22,23,24]. In our model, the effects of HKII silencing are consistent with a contribution of this enzyme to cell survival, while the effects of apigenin on several metabolic proteins suggest a broader response. Pharmacologic approaches to HKII and glycolysis have been investigated in other cancer models [25].

The xenograft results extend the cellular observations: apigenin-treated tumors were smaller and showed changes in glycolysis-associated and cell death markers. Body weight and general appearance did not reveal obvious treatment-related changes during the 24-day observation period, but these measures alone cannot establish systemic safety. Xenografts also differ from human primary tumors in immune function, stromal composition, implantation site, and growth kinetics. The 20 mg/kg apigenin dose is consistent with that used in a prior MM model [26]. An increased Bax/Bcl-2 ratio and cleaved caspase-3 in the apigenin-containing groups support an apoptotic response; however, caspase-9 activation was not measured in these tumors, so the specific caspase-9–caspase-3 sequence remains to be established.

The lactate-acclimated cells used here were previously reported to differ from their parental lines in glycolytic features and gemcitabine response [19]. In the present experiments, HKII silencing was associated with lower PFKP and PDH protein levels and lower measured HK and PDH activities (Figure 5D,E). The reduction in PDH activity may reflect the decrease in PDH protein abundance or a broader metabolic response following HKII depletion; these data do not show that HKII directly regulates the catalytic activity of PDH. Resolving that mechanism would require additional measurements, including PDH activity relative to PDH protein abundance and assessment of its regulatory state. Apigenin also decreased OXPHOS protein levels, whereas HKII knockdown did not show the same effect, supporting a distinction between their actions. Changes in p-AKT (Ser473) and p53 accompany these responses, but their causal position relative to HKII has not been tested. AKT and p53 are known to influence glucose metabolism and mitochondrial survival pathways [27,28,29,30,31]; HKII has also been linked to AKT-related signaling in cervical cancer and in quercetin-treated hepatocellular carcinoma models [32,33]. However, feedback among these signals remains a possibility rather than a demonstrated mechanism in the present study.

HKII knockdown and apigenin treatment were associated with mitochondrial depolarization and increases in cleaved caspase-3 and other cell death markers. These observations are compatible with mitochondrial involvement in apoptosis and with prior reports of HKII-dependent protection of the mitochondrial outer membrane [27,29]. Studies in lymphoma and hepatocellular carcinoma have also linked HKII to tumor-cell growth or glycolysis [34,35]. We did not measure cytochrome c release, caspase-9 cleavage, or the effect of inhibiting this pathway. Thus, the data support apoptosis but do not directly establish the full intrinsic caspase-9–caspase-3 cascade. The observed G2/M changes may also accompany the loss of viability, although the signals connecting cell cycle changes, p53, and p-AKT (Ser473) were not defined here. Other cancer models have implicated ERK1/2/CREB/BCL-2 signaling in the regulation of mitochondrial apoptosis [36]. Further experiments are needed to determine how HKII, mitochondrial function, and these signaling proteins interact in apigenin-treated MM cells.

## 5. Conclusions

In lactate-acclimated MM models, apigenin reduced glycolysis-associated proteins and cellular energy measures and increased markers of apoptosis and necroptosis. HKII silencing produced partly overlapping effects, supporting an association between HKII-dependent metabolism and cell survival. The direct target of apigenin and the precise mitochondrial apoptotic sequence, including caspase-9 activation, remain unresolved. These findings warrant further mechanistic and preclinical evaluation of apigenin in MM.

## Data Availability

The data supporting the reported findings are presented within the article.
